# The dynamic developmental process of adolescent non-suicidal self-injury disclosure: a qualitative study

**DOI:** 10.3389/fpsyt.2025.1613725

**Published:** 2025-10-31

**Authors:** Zikang Liu, Ruofei Wang, Xingxue Li, Zhe Yuan, Tong Wu, Hong Liang

**Affiliations:** ^1^ Department of Psychology, Chengde Medical University, Chengde, China; ^2^ Beijing Suicide Research and Prevention Center, Beijing Huilongguan Hospital, Beijing, China

**Keywords:** adolescents, non-suicidal self-injury, self-disclosure, self-injury, qualitative study

## Abstract

**Background:**

Non-suicidal self-injury poses a serious threat to adolescents’ mental health, and if such behavior remains unexpressed or unshared over a long period, it may lead to further deterioration of their psychological well-being. The public lacks a comprehensive understanding of the ways in which adolescents express non-suicidal self-injury.

**Methods:**

Guided by the principle of intensity sampling, this study selected cases with high information richness and intensity through snowball sampling, referral sampling, and opportunistic sampling methods. Seventeen adolescents meeting the DSM-5 criteria for non-suicidal self-injury were recruited from psychiatric outpatient clinics as study participants. Using a grounded theory approach based on in-depth interviews, a theoretical model of adolescent non-suicidal self-injury disclosure behaviors was gradually constructed.

**Results:**

A total of 132 concepts, 51 categories, 23 subcategories, and 7 core categories were identified in this study. The core categories in the dynamic developmental process of adolescent non-suicidal self-injury disclosure include shame, emotional experience, feedback response, outcome adjustment, disclosure motivation and expectation, disclosure behavior, and contextual factors. Centered on the core category of “the dynamic developmental process of adolescent non-suicidal self-injury disclosure,” a four-stage theoretical model of dynamic adaptation in adolescent non-suicidal self-injury disclosure was constructed. This model, starting from shame and situational triggering events, delineates a four-stage dynamic process of adolescent non-suicidal self-injury disclosure: in the situational trigger stage, shame suppresses the willingness to disclose; during the disclosure decision-making stage, adolescents weigh disclosure motives against expectations and emotional experiences, generating disclosure conflicts; in the disclosure action stage, adolescents select specific methods and targets for NSSI disclosure, receiving varying feedback; and in the adjustment and adaptation stage, positive feedback promotes help-seeking, whereas negative feedback reinforces avoidance.

**Conclusions:**

The dynamic four-stage theoretical model of adolescent non-suicidal self-injury disclosure developed in this study clearly delineates the stage-specific, cyclical, and feedback-driven characteristics of NSSI disclosure. This model innovatively reveals how adolescents continuously navigate psychological trade-offs and adjust their behaviors between shame, situational triggers, disclosure motives, and emotional experiences during the NSSI disclosure process, offering a novel theoretical perspective for understanding NSSI disclosure. Simultaneously, this model can be applied to design personalized support strategies for different stages of self-injury disclosure, enhance the effectiveness of psychological interventions, and reduce the risk of adolescent self-injury.

## 1 Introduction

Non-suicidal self-injury (NSSI) is an issue that deserves widespread attention across society. NSSI has become prevalent among adolescent populations, and its incidence has shown a trend of increasing year by year (1). In China, the prevalence of NSSI among adolescents is 27.4%, which is higher than the global prevalence of NSSI among adolescents (2, 3). It has become a widely recognized major public health issue affecting the physical and mental health of adolescents (4).

Current research does not yet provide a clear definition of NSSI disclosure. Chinese scholar defines NSSI disclosure as the act of an individual voluntarily revealing their NSSI behaviors to others, regardless of the underlying motivations (5). Some scholars also argue that self-disclosure is an important signal of self-help-seeking behavior among NSSI patients (6). Western scholars believe that disclosing information is the first step towards the prevention and intervention of NSSI (7). The definition of NSSI disclosure in this study is as follows: Adolescents who engage in NSSI often share their self-injurious behaviors with others for specific purposes and receive feedback, either having done so in the past or currently doing so.

Adolescents are in the puberty stage, during which their self-disclosure function is naturally low (8). As a result, many adolescents are unwilling to disclose their NSSI behaviors to others. Additionally, NSSI itself is highly covert (9), which makes it difficult to detect many instances of NSSI among adolescents, thereby hindering effective early identification and intervention. If adolescents do not disclose their NSSI behaviors to others for an extended period, they may remain trapped in feelings of guilt, sorrow, and depression, which could even exacerbate suicidal thoughts (10). Research suggests that disclosing self-injurious behaviors is the first step in early identification of NSSI, and disclosure of self-harm may provide opportunities for early intervention in self-injury and suicidal behaviors (11, 12). It is evident that promoting the disclosure of NSSI in adolescents and identifying and recognizing NSSI earlier has become an urgent issue that needs to be addressed (13).

Although existing research has made some progress in adolescent NSSI disclosure, it is largely limited to examining the presence and frequency of disclosure, the measurement methods of disclosure, and its influencing factors, typically employing questionnaire surveys or thematic analysis (14–16). NSSI disclosure is important. However, most existing studies assess self-injury disclosure from a static perspective, lacking a systematic understanding of its stage-specific, cyclical nature and underlying psychological trade-off mechanisms. Particularly regarding dynamic changes and feedback mechanisms, the academic field still lacks theoretical models to elucidate how adolescents continuously adjust their self-injury disclosure behaviors across different contexts. According to Jourard’s self-disclosure theory (17), disclosure is an interactive behavior in which individuals adjust the content and depth of their disclosure based on others’ responses, suggesting that NSSI disclosure should also be understood as a dynamic, continuously feedback-driven process.

Currently, research on the disclosure behaviors of NSSI in adolescents is still relatively limited. This study aims to enhance a comprehensive understanding of adolescent NSSI disclosure behaviors, enrich relevant theoretical frameworks, and provide scientific evidence and practical guidance for the early identification and intervention of NSSI in the future.

## 2 Materials and methods

### 2.1 Design

This study employed the analytical procedures of grounded theory. This process utilized NVIVO12 for coding; however, the coding was fundamentally researcher-driven, relying on the researchers’ expertise and theoretical competence rather than the software’s automated functions. First, open coding was conducted, analyzing the transcripts line by line, labeling initial concepts, and continuously comparing them to form preliminary categories. Subsequently, during the axial coding phase, these categories were further integrated, the relationships among them were explored, and connections between conditions, interactions, and outcomes were established. In the selective coding phase, core categories were gradually aggregated, and the various subcategories were integrated with the core categories to construct the theoretical model. Throughout the analysis process, the research team continuously conducted comparative analyses to ensure that the constructed category system possessed stability and explanatory power. Simultaneously, memos were written to document reflections, hypotheses, and analytical processes concerning concepts, categories, and their interrelationships.

### 2.2 Participants

Adolescents who received outpatient treatment and met the fifth edition of the Diagnostic and Statistical Manual of Mental Disorders (DSM-5) diagnostic criteria for NSSI between February 2024 and October 2024 were selected (18). Guided by the principle of intensity sampling, this study selected cases with high information richness and intensity through snowball sampling, referral sampling, and opportunistic sampling methods. At the initial stage of the study, the number of interviewees was not predetermined; instead, the interview process was guided by the data saturation principle of grounded theory (19). During implementation, researchers conducted grounded theory coding and analysis concurrently with the interviews to ensure timely identification and comprehension of key themes and core concepts within the interview data. When interviews no longer yield new information or themes, data is considered to have reached theoretical saturation, and the interviews are concluded (20). Ultimately, theoretical saturation was reached after interviewing the 17th participant. Subsequently, two additional participants were interviewed for verification, and no new insights were identified, ensuring that the data had indeed reached saturation. Therefore, the final number of interview participants in this study was 17, including 6 males and 11 females, with a mean age of 14.94 ± 1.56 years. The basic information is presented in **Table 1**.

**Table 1:** Basic Information of the participants.

Code	Gender	Age	Place of residence	Code	Gender	Age	Place of residence
Participant 1	Female	12	Urban	Participant 10	Female	15	Urban
Participant 2	Female	16	Rural	Participant 11	Male	15	Urban
Participant 3	Female	13	Urban	Participant 12	Male	17	Rural
Participant 4	Female	16	Rural	Participant 13	Female	15	Urban
Participant 5	Male	14	Urban	Participant 14	Female	18	Urban
Participant 6	Female	15	Rural	Participant 15	Female	14	Urban
Participant 7	Male	15	Rural	Participant 16	Male	17	Rural
Participant 8	Male	14	Urban	Participant 17	Female	13	Urban
Participant 9	Female	15	Rural				

### 2.3 Ethical statement

This study was approved by the Institutional Review Board of Beijing Huilongguan Hospital (2024-51-Science). Prior to the interview, the researchers provided the adolescents and their guardians with a detailed explanation of the study’s purpose, procedures, and privacy protection measures. Informed consent was obtained from both the adolescents and their guardians, followed by the signing of consent forms. Throughout the interview process, participants’ identities were kept strictly confidential. All data were anonymized by replacing real names with identification codes, and any information that could potentially reveal personal identities was removed. Audio recordings of the interviews were used solely for research analysis, and both the recordings and transcriptions were subject to strict access control, available only to members of the research team. The transcribed texts were used for data analysis after de-identification, while the original audio recordings were securely preserved and stored in encrypted form.

### 2.4 Data collection

This study collected interview data through in-depth interviews, which were conducted in the psychotherapy room of the hospital outpatient clinic (21). To minimize the potential impact of gender on respondents’ answers and to ensure the protection of minor participants, the interview team consisted of one male and one female interviewer (22). Both interviewers were master’s degree students in clinical psychology and counseling, with one of them holding a qualification as a psychotherapist. Each interview lasted 30–40 minutes. After the interview, the audio recordings were organized and transcribed within 24 hours, converting the audio content into written text to ensure the integrity and timeliness of the data.

The interview outline for this study was designed by integrating the results of literature review and expert opinions. The first draft of the interview outline was based on the theoretical and empirical research framework from extensive literature and was submitted to a senior expert in the field of psychology for review and revision. Prior to the formal interviews, we conducted preliminary interviews with two adolescents. Based on their feedback and responses, and under the guidance of experts, the research team held multiple internal discussions to repeatedly revise the interview outline. The final version of the interview outline is as follows: 1) Under what circumstances would you disclose your self-harming behavior to others? 2) How do you feel after disclosing your self-harming behavior to others? 3) What was the feedback from others after you disclosed your self-harming behavior? 4) After disclosing your self-harming behavior, what kind of feedback do you hope to receive from others? 5) What was the initial purpose or motivation for disclosing your self-harming behavior?

### 2.5 Data analysis

This study uses grounded theory as the primary method for data analysis, with the aim of further developing relevant theories. Grounded theory is a qualitative research method, characterized by generating theories from raw data through a systematic analysis process, rather than relying on existing theoretical frameworks for verification (23). This method is exploratory in nature, emphasizing the “groundedness” of the theory, meaning that the generated theory should be closely connected to the data from the research context. Under the guidance of grounded theory, the researcher engages in continuous data interaction and dynamic analysis, using methods such as open coding, axial coding, and selective coding to deconstruct, categorize, and integrate the data. After each interview, the researchers immediately performed preliminary coding of the data, identified key concepts, and compared them with existing codes and categories, continuously refining the data collection strategy to obtain more information-rich data. When discrepancies arose in coding, the research team members engaged in discussions until consensus was reached, consulting the original data and memo-recorded analytical reflections as needed to ensure coding reliability and consistency. Through this step-by-step analytical process, the researcher is able to conduct in-depth comparisons between different data, discern their similarities and differences, identify patterns and relationships within the data, and extract concepts and categories with theoretical significance.

## 3 Results

### 3.1 Open coding

In qualitative research, open coding is the first level of coding in grounded theory (24). In this study, for example, the phrase “I hope they give me some feedback that makes me feel comfortable” (Respondent 13) is coded as “hoping for a comfortable feedback” (concept label); these concept labels are repeatedly compared and then categorized into broader themes. For example, concept labels such as “hoping for a comfortable feedback” and “hope to receive care” were coded into the category “desire for positive feedback.” This study established a total of 132 concepts (A1-A132) and 51 categories (B1-B51). see **Table 2**.

**Table 2:** Open coding results.

Basic categories	Conceptualization
B1 Covering the wound with clothes	A1 put on an outer garment A2 Wrap oneself up A3 Wear sun protection A4 Wear long sleeves A5 Wear knee-length clothing
B2 Conceal self-harm	A6 Don’t want them to know A7 Do not disclose self-harm to them
B3 Feelings of shame and guilt toward others	A8The parents of my classmates make me feel ashamed and guilty A9 Parents induce feelings of shame and guilt in oneself
B4 Affect work	A10 Afraid it will affect future job prospects A11 Feel that finding a job is difficult
B5 Be discriminated against	A12Afraid of being discriminated against by others A13 Being viewed differently by others
B6 Be mocked	A14 New skin A15 My first impression was that I was being mocked A16 Clean arms look beautiful A17 Speak ill of
B22 Emotionally agitated	A55 Having unexpected emotional responses A56 Keep crying A57 Break down
B23 Parents do not understand	A58 Thinks studying is important A59 More of a lack of understanding A60 The first reaction is not understanding
B24 Indifference	A61 Cut if you want A62 Doesn’t care about me A63 Thinks it’s not a big deal A64 Have never comforted anyone
…	…
B39 Hopes for verbal comfort	A102 Says comforting words A103 Comfort oneself more through words
B40 Hope to be engaged in activities of personal interest	A104 Takes me out to have fun A105 Buys me bubble tea
B41 Hope to receive positive feedback	A106 Hoping for a comfortable feedback A107 Hope to receive care
B42 Hopes for a hug	A108 Hope to give me a hug A109 Hope to be hugged
B43 Hopes to take away self-harm tools	A110 Hopes to have my razor blade taken away A111 Hope someone would take away my utility knife
B44 Waiting to be discovered	A112 Accidentally seeing it A113 See if they can guess it A114 Subtly bring it up A115 Accidentally being seen
B45 Not actively revealing	A116 It’s impossible to bring it up with them A117 Never told them
B46 Conceal oneself	A118 Hides it deeper A119 Pretends to be like a normal person A120 Hopes it is never discovered by others
B47 Revealing to peers	A121 Expressing it to someone of similar age A122 Telling a friend
B48 Being discovered at home	A123 Sister finds out A124 While taking a shower A125 Going for a walk A126 Taking clothes off
B49 Easily exposed in summer	A127 When wearing short sleeves A128 While swimming in summer
B50 Exposed during a visit to the hospital	A129 Asked about self-harm during a doctor visit A130 Discovered by mother at the hospital
B51 When going out with friends	A131 Taking public transport with a friend A132 Friend actively discovers it

The open coding presented in the table represents a selected portion of the codes.

### 3.2 Axial coding

Axial coding is the second-level coding process in grounded theory, aiming to further analyze the categories identified during the open coding phase and to explore and reveal the internal relationships among them (25). see **Table 3**.

**Table 3:** Axial coding results.

Primary category	Subcategory	Basic categories
D1 Shame	C1Ways to cope with shame	B1 Covering the wound with clothes
		B2 Conceal self-harm
	C2 Public shame	B3 Feelings of shame and guilt toward others
		B4 Affect work
		B5 Be discriminated against
		B6 Be mocked
	C3 Personal stigmatization	B7 Shame in displaying the wound
D2Emotional experience	C4 Emotional relief	B8 Feel comfortable and warm
		B9 Be understood
	C5 Negative emotional experience	B10 Vicarious suffering
		B11 Unable to take in what others say
		B12 Not being understood
		B13 Sense of powerlessness
		B14 Increase the burden
		B15 Guilt and regret
	C6 Alleviating the pressure of hiding self-harm	B16 Feel relieved after disclosing self-harm
D3 Feedback response	C7 Behavioral persuasion	B17 Recommend seeking medical attention
		B18 Monitor and prevent self-harm
	C8 Receiving care and love	B19 Care and comfort
	C9 Exercising teacher duties	B20 Notify the parents
	C10 Family members have mood swings	B21 Guilt and self-hatred
		B22 Emotionally agitated
	C11 Criticism under negative attitudes from family	B23 Parents do not understand
		B24 Indifference
		B25 Sarcasm and mockery
		B26 Excessive blame
	C12 Father provides support	B27 Father reduced pressure
		B28 Father’s care and affection toward oneself
	C13 Escaping reality	B29 Parental avoidance
D4 Outcome adjustment	C14 Parents realize the seriousness of the issue	B30 Thinks the child is ill
		B31 Talk to the child
	C15 Changes in family relationships and parenting styles	B32 Deterioration of family relationships
		B33 Strengthened supervision and monitoring
	C16 Self-harm cycle	B34 Continued self-harm
		B35 Worsening self-harm
	C17 Reduction in self-harm	B36 Change in methods of self-harm
		B37 Gradually stopped self-harming
D5 Motivation and expectations for disclosure	C18 Receiving emotional support and understanding	B38 Hopes for perspective-taking
		B39 Hopes for verbal comfort
	C19 Taking practical actions	B40 Hope to be engaged in activities of personal interest
		B41 Hope to receive positive feedback
		B42 Hopes for a hug
		B43 Hopes to take away self-harm tools
D6 Disclosure behavior	C20 Subtle expression	B44 Waiting to be discovered
	C21 Refusing to reveal	B45 Not actively revealing
		B46 Conceal oneself
	C22 Actively revealing	B47 Revealing to peers
D7 Situational factors	C23 Situations of revealing self-harm	B48 Being discovered at home
		B49 Easily exposed in summer
		B50 Exposed during a visit to the hospital
		B51 When going out with friends

### 3.3 Selective coding

Selective coding is the third-level coding process in grounded theory, through which seven core categories were ultimately identified. The seven core categories are: D1 shame; D2 emotional experience; D3 feedback response; D4 outcome adjustment; D5 disclosure motivation and expectation; D6 disclosure behavior; and D7 contextual factors. see **Table 3**.

### 3.4 Construction of the theoretical model

Centered on the core category of “the dynamic developmental process of adolescent NSSI disclosure,” this study progressively constructed a four-stage dynamic adaptation model of adolescent NSSI disclosure through continuous comparative analysis and theoretical abstraction. During the open coding stage, a wide range of concepts related to NSSI disclosure, including shame, expectation, and others’ reactions, were identified. In the axial coding stage, these concepts were integrated into several core categories, revealing the underlying logical chain of NSSI disclosure behavior. In the subsequent process of selective coding and storyline integration, the research team found that these categories did not exist in isolation but formed a dynamically evolving process aligned with temporal sequences and psychological trajectories.

This process was illustrated through a storyline (26): After engaging in NSSI behaviors, adolescents often experience a sense of shame, which encompasses both self-perceived shame and shame imposed by societal attitudes. Adolescents may also choose not to disclose their self-injurious behaviors to others in an effort to reduce feelings of shame. Adolescents’ expectations regarding the outcomes of disclosure and their motivations at the time of disclosure play a critical role in determining whether they choose to disclose their self-injurious behaviors. Many adolescents hold positive motivations and expectations when disclosing self-injury. They often hope to seek help, gain understanding, and receive care through the act of disclosure. However, there is often a conflict between adolescents’ expectations of disclosing self-injury and the reality of the disclosure experience. Adolescents are afraid of being misunderstood by others. Emotional experiences following disclosure also influence the manner in which adolescents choose to disclose self-injury. When adolescents experience intense negative emotions after disclosing self-injury, it can hinder further disclosure. Conversely, when they experience predominantly positive emotions, it can facilitate further disclosure. When adolescents choose to disclose self-injury, they may adopt one of several approaches: direct disclosure, indirect expression, or deliberate concealment. After disclosing self-injury, the feedback from others is crucial for adolescents. If the feedback is positive, adolescents are more likely to accept help. Conversely, if the feedback is negative, adolescents may reject further disclosure and even experience increased psychological distress.

**Figure 1:**
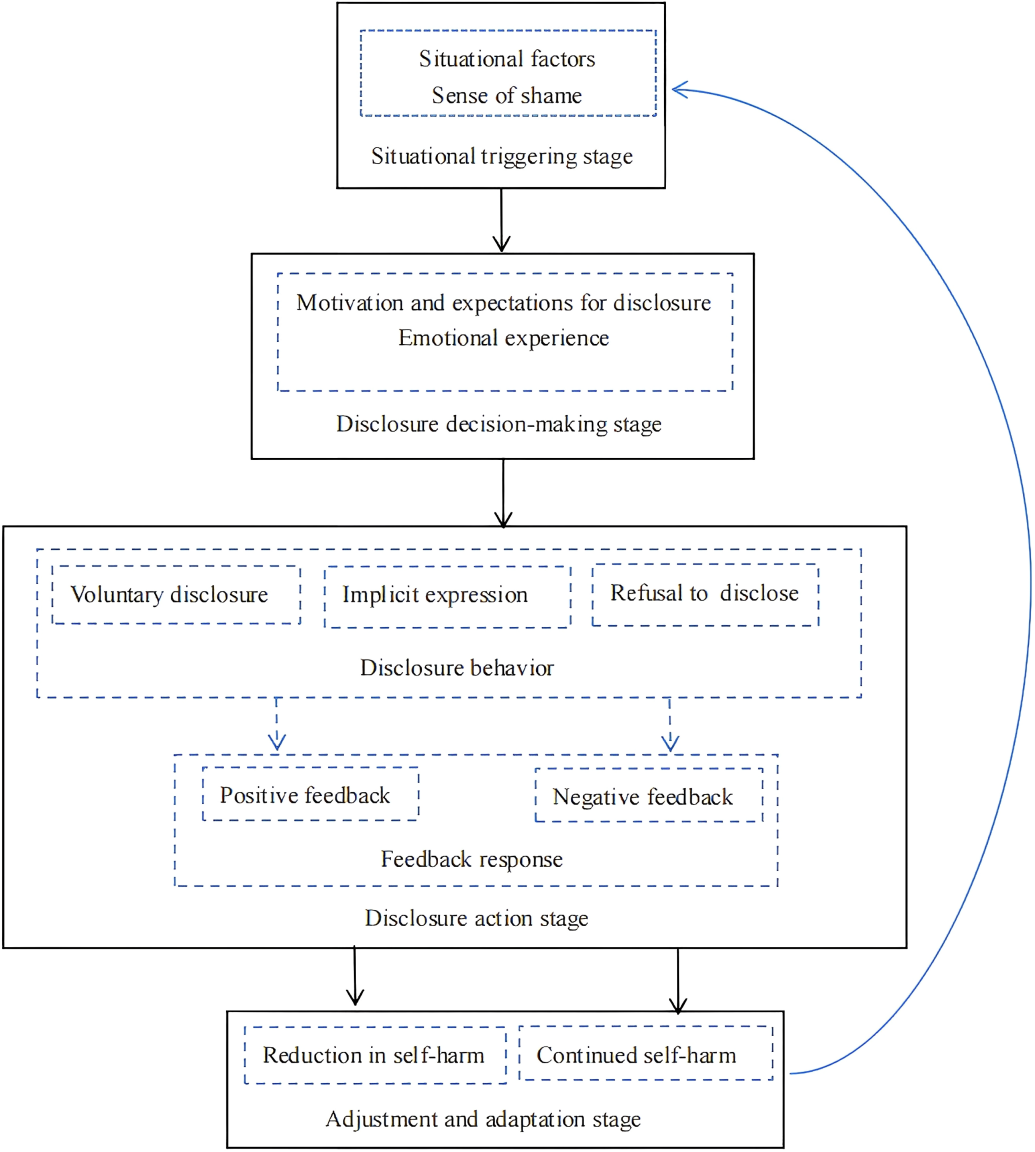
Dynamic adaptation four-stage theoretical model of adolescent non-suicidal self-injury disclosure.

Based on the aforementioned storyline, the following theoretical model can be derived. This theoretical model is named the “Four-Stage Dynamic Adaptation Model of Adolescent Non-suicidal Self-Injury Disclosure.” It serves to explain the process of adolescent NSSI disclosure. Specifically, adolescents’ NSSI disclosure often begins with particular contextual triggers (D1 shame and D7 contextual factors). The core mechanism at this stage lies in the inhibitory effect of shame on the willingness to disclose under stressful circumstances. Subsequently, adolescents enter the disclosure decision-making stage (D2 emotional experience and D5 disclosure motivation and expectation). At this stage, adolescents balance their motivations and expectations with their emotional experiences, which often generates internal conflict about whether to disclose. In the disclosure action stage (D3 feedback response and D6 disclosure behavior), adolescents choose among three strategies: voluntary disclosure, implicit expression, and refusal to disclose, with their decisions shaped by both anticipated reactions and actual feedback. Finally, adolescents enter the adjustment and adaptation stage (D2 emotional experience and D4 outcome adjustment) based on the feedback received after disclosure, forming either promotive or avoidant adaptation patterns. The four-stage structure of the model emerged from repeated comparison and integration of interview data, reflecting the temporal evolution of adolescent NSSI disclosure behaviors and illustrating the spiral-like process of adolescents’ psychological adaptation. See **Figure 1**.

### 3.5 Shame

In the situational triggering stage of the model, shame serves as a key mechanism that inhibits adolescents’ willingness to disclose their NSSI behaviors. Adolescents often perceive showing self-injury scars to others as a source of shame. They regard these marks as something that should be hidden from others. This self-perceived sense of shame hinders adolescents from disclosing their self-injurious behaviors to others. For adolescents engaging in NSSI, the sense of shame primarily stems from concerns about how their peers’ parents perceive their self-injurious behavior. They worry about being mocked and stigmatized by both parents and peers within the school environment. They fear that disclosing self-injury will lead to being viewed differently by their classmates.

Adolescents avoid disclosing their self-injury due to feelings of shame:

“*I feel some guilt, and I think it’s shameful to display this in front of them.*” (Participant 2)

Emphasize that the attitudes of peers and parents intensify the sense of shame:

“*I think they would go home and tell their son not to live like me, and I feel ashamed*.” (Participant 6)

Reflects underlying concerns about potential discrimination:

“*I’m also afraid of being discriminated against. The teachers don’t know about my self-harm behavior*.” (Participant 8)

This reveals the inhibitory role of shame in the situational triggering stage on the willingness to disclose self-injury, providing a psychological backdrop for subsequent disclosure actions.

### 3.6 Contextual factors

Situational factors and feelings of shame jointly constitute the situational triggering stage of the model. Adolescents typically disclose their NSSI behaviors when they are observed by others in specific situations. For example, in hot summer months, wearing short sleeves may expose scars. Alternatively, at home, self-injurious behaviors may be discovered by family members. Some adolescents may unintentionally expose their self-injury while hanging out with friends. Before disclosing NSSI behaviors, the combined influence of shame and contextual factors affects adolescents’ disclosure of their NSSI behaviors.

The passive triggering of self-injury disclosure by situational factors:

“*One summer, while riding the bus in a short-sleeved shirt, my friend noticed bruises all over my body and began asking me how I got like that*.” (Participant 1)

Non-volitional disclosure within the family context:

“*At that time, I was about to take a shower at home, and when I undressed, my mother noticed*.” (Participant 15)

In the situational triggering stage of the model, situational factors interact with shame, serving as key external conditions influencing adolescents’ willingness to disclose NSSI.

### 3.7 Disclosure motivation and expectation

Adolescents typically disclose self-injury to seek understanding and support rather than discipline. This motivation and expectation constitute a significant internal driving force in the disclosure decision-making stage of the model. The motivation for adolescents to disclose self-injury is to relieve their emotions and gain attention and understanding from others, rather than to receive discipline from others. Adolescents disclose NSSI behaviors with a greater expectation of receiving support and understanding from others. In peer relationships, adolescents hope that their friends will provide more care and understanding. For parents, adolescents expect them to adopt an empathetic perspective, understanding their emotional distress and the underlying reasons for self-injury, rather than merely increasing surveillance and expressing concern. After disclosing NSSI behaviors, adolescents typically expect others to take concrete actions to prevent further self-injury. These actions include caring behaviors from friends or family, such as offering hugs, involving the adolescent in activities they enjoy, and providing more attentive care. Additionally, some adolescents hope that their parents will adjust or change their parenting style to adopt a more understanding and accepting attitude. Overall, the motivations and expectations behind adolescents’ disclosure of NSSI behaviors are typically positive, with adolescents being more inclined to seek positive support from others.

The motivation for disclosure lies in emotional relief:

“*Actually, I disclosed my self-injury not to be scolded or controlled by my parents, but because I really couldn’t hold it in anymore, and saying it made me feel somewhat relieved*.” (Participant 2)

The motivation for disclosure lies in seeking help:

“*I told my friends about my self-injury to get their help; I couldn’t control my self-injury, but I also didn’t want to sink deeper into it*.” (Participant 16)

Expectation of receiving practical support:

“*What I hope for is that they take away my razor blades, and once they take them, they prevent me from continuing to use them. I hope they take action, rather than just giving verbal lectures, because anyone can speak verbally*.” (Participant 5)

Expectation that others engage in perspective-taking to understand their true thoughts:

“*I want them to consider my thoughts from my perspective, but they only think about their own perspective*.” (Participant 4)

Expectation of positive emotional feedback:

“*I want others to care about me, or give me some feedback that makes me feel comfortable*.” (Participant 8)

This category reveals how disclosure motivations and expectations influence adolescents’ willingness to disclose and their choice of disclosure methods during the decision-making stage of the model.

### 3.8 Emotional experience

In the disclosure decision-making stage of the model, adolescents weigh their motivations and expectations against their emotional experiences, resulting in disclosure conflict. After disclosing NSSI behaviors to friends, whether voluntarily or passively due to external factors, adolescents typically experience positive emotions. Adolescents with NSSI often feel comfort and warmth after disclosing self-harm to friends, believing that the care and understanding from friends help alleviate inner pain. This emotional support also prompts adolescents to reflect on whether they should engage in self-harm. When adolescents disclose NSSI behaviors to family members, the primary recipients of this disclosure are usually their parents. After disclosure, adolescents often experience feelings of guilt and regret, accompanied by a strong sense of helplessness. These emotional experiences may further affect the disclosure of NSSI behaviors and even exacerbate self-harming actions.

Positive emotional experiences facilitate the disclosure of self-injury:

“*I have a best friend who has been with me for five years. When he cares for me, I feel so happy because he cares about me. I believe he is the one who truly understands me*.” (Participant 1)

Negative emotional experiences may undermine the disclosure of self-injury:

“*I am afraid of adding burden to them. After revealing this, I still feel guilty and regretful, and even though I received care from my brother, this guilt hasn’t faded at all*.” (Participant 4)

This category reveals the crucial role of emotional experiences in modulating adolescents’ intentions to disclose self-injury during the decision-making stage of the model.

### 3.9 Disclosure behavior

After experiencing inner conflict and struggle, adolescents disclose their NSSI behaviors in various ways. Typically, these disclosure methods can be categorized into three types: active disclosure, implicit expression, and refusal to disclose. Active disclosure refers to adolescents directly talking to others about their self-harm behaviors, but the recipients of active disclosure are generally peers and close friends. Implicit expression refers to adolescents hinting at their self-harm behaviors in an indirect manner or waiting for others to discover the self-harm. Refusal to disclose refers to individuals deliberately concealing their self-harm behaviors and not mentioning them to anyone. Adolescents disclose self-injury through both active disclosure and implicit expression, and the feedback they receive significantly influences their future disclosure behaviors.

Implicit expression:

“*I subtly mentioned my self-injury to see if they could figure it out*.” (Participant 4)

Implicit expression:

“*I didn’t hide my self-injury, but I also didn’t proactively tell others; I just waited for someone to notice*.” (Participant 7)

Active disclosure:

“*I would proactively tell peers of similar age that I engage in self-injury.*” (Participant 16)

This category presents three distinct disclosure behaviors, forming the core behavioral patterns in the disclosure action stage of the model.

### 3.10 Feedback response

In the disclosure action stage of the model, adolescents select different disclosure behaviors based on the target audience and receive corresponding feedback. When family members learn of an adolescent’s NSSI behaviors, the feedback is often negative. Parents typically display strong emotional reactions and may criticize and reprimand the adolescent. From the father’s perspective, although he also feels shocked and helpless, in certain situations, he may attempt to provide some degree of support, such as reducing pressure and feeling sympathy for his child. In addition, parents may adopt avoidance strategies when facing this reality. For example, mothers may refuse to acknowledge their child’s mental health issues, while fathers may cope with their own stress through behaviors like smoking.

Reflects how parental criticism exacerbates psychological distress:

“*My mom never comforted me; instead, it felt more like criticism. You could say there must be a reason behind what we do. Not to mention self-harming, even being doubted or criticized by others regularly is uncomfortable, right? Why can’t we talk things through calmly, instead of getting into an argument? Does arguing solve the problem?*” (Participant 2)

Illustrates the father’s avoidance strategies:

“*My dad might feel a bit guilty, he always goes outside to smoke to escape.*” (Participant 9)

Illustrates the mother’s avoidance strategies:

“*They think I don’t have this illness at all, and then my mom subconsciously believes it’s not an illness, so no one pays attention to me.*” (Participant 5)

Compared to feedback from the family, adolescents often receive more positive feedback from friends or classmates. Adolescents receive discouragement, comfort, and care from peers, thereby gaining social support to some extent. For teachers, feedback is more often based on professional responsibility, such as informing parents and taking appropriate measures to discourage adolescents’ self-harming behaviors. Feedback from these different social roles not only affects adolescents’ emotional experiences but may also further shape the trajectory of their future disclosure behaviors and the development of self-harming actions.

Indicates that peer support provides positive feedback:

“*My friend often comforts me, saying ‘What’s wrong with your nose?’ I felt a bit bitter hearing that, but I also felt that he really cares about me.*” (Participant 13)

Teachers intervene through actions to discourage self-injury:

“*The teacher told me to stop engaging in this behavior and also took away my knife.*” (Participant 1)

Teachers intervene through actions to discourage self-injury:

“*It was not discovered by my family; it was the teacher who found out and then informed my parents. The teacher told my parents about my situation.*” (Participant 9)

Disclosure behaviors and feedback responses constitute the disclosure action stage of the model. In this stage, adolescents choose different disclosure behaviors, and feedback from various social roles shapes their future disclosure actions, influencing the trajectory of their self-injurious behaviors.

### 3.11 Outcome adjustment

In the outcome adjustment stage of the model, following the disclosure of self-injury, adolescents and their families undergo a series of adjustments. After adolescents disclose NSSI behaviors, the family typically undergoes a series of changes. These changes are primarily reflected in the increased supervision of children by parents, the gradual modification of parenting styles as self-harm behaviors are disclosed, and the increasing awareness of children’s mental health issues. To prevent the recurrence of self-harming behaviors in children, parents often implement stricter supervisory measures. At the same time, parents tend to view their child’s behavior as a manifestation of mental health issues and attempt to engage in more communication to gain a deeper understanding of their child’s inner state and needs. However, the communication methods employed by parents are those that the child does not accept. Parental reinforcement of supervision:

“*I was always staying at home, and then they insisted on taking me out for a walk. During the walk, he saw me, and then my parents started watching me closely, not allowing me to self-harm.*” (Participant 7)

Attempts at communication and interaction:

“*Later, whatever I did, they wanted to know what I was doing, always feeling like something was wrong with me, insisting on coming over to talk to me. They never understood why I self-harmed and always wanted to have a conversation with me.*” (Participant 11)

This process reflects the adaptation of the family system and the adjustments of the adolescent following self-injury disclosure, forming the core mechanism of the model’s outcome adjustment stage.

## 4 Discussion

The dynamic adaptation theoretical model of adolescent NSSI disclosure developed in this study reveals, from a dynamic perspective, that the disclosure of NSSI in adolescents is an evolving and changing process. The model comprises four interrelated stages. The first is the situational trigger stage, during which adolescents experience an impulse to disclose under the influence of external environmental factors and feelings of shame. The second stage is the disclosure decision-making stage, in which adolescents experience an internal conflict between their motivations and expectations for disclosing self-injury and the emotional experiences they undergo. The third stage is the disclosure action stage, during which adolescents choose different modes of disclosure, such as explicit disclosure, implicit expression, or refusal to disclose. Following disclosure, the nature of the feedback plays a crucial role in shaping subsequent developments. The final stage is the outcome adjustment stage. If adolescents receive positive feedback, they are more likely to seek further help and may exhibit a reduction in self-injurious behaviors. However, if the feedback is negative, adolescents may continue or even intensify their self-injurious behaviors, becoming trapped in a vicious cycle. Within this overarching framework, adolescents’ motivations for disclosing self-injury and their expectations regarding disclosure outcomes jointly influence the occurrence and progression of NSSI disclosure, which unfolds as a dynamic process that continually evolves under the influence of external feedback.

According to Jourard’s theory of self-disclosure, individuals sustain interpersonal relationships and maintain psychological well-being by expressing their authentic feelings to others (17). Self-disclosure is not only the foundation for establishing close relationships but also an important means for emotional regulation and psychological growth. For adolescents engaging in non-suicidal self-injury, self-disclosure is equally significant. On one hand, disclosing self-injury can help adolescents gain understanding and support from others, thereby alleviating their distress. On the other hand, this disclosure process carries the risk of rejection, stigmatization, or being misunderstood by others. Therefore, adolescents often fluctuate and hesitate when deciding whether to disclose their self-injury experiences. Based on Jourard’s self-disclosure theory, self-disclosure is not only a crucial aspect of adolescents’ psychological adaptation to self-injury but also a key factor influencing the establishment of their support systems (17).

The results of this study indicate that shame and situational factors together constitute the situational trigger phase of adolescent NSSI behavior disclosure. Adolescents generally view NSSI as a strategy for emotion regulation (27). However, self-harm leaves scars, which lead to feelings of shame and concern. As a result, adolescents are typically reluctant to openly disclose their self-harming behaviors and tend to conceal or hide them (28). Furthermore, this study found that the disclosure of adolescent self-harming behaviors often occurs in specific contexts, such as particular social settings, hot summer weather, and when wearing fewer clothes. In these contexts, scars are more likely to be noticed by others, triggering feelings of shame and anxiety about social evaluation. The interaction between contextual factors and feelings of shame places adolescents in a psychological dilemma prior to disclosing NSSI behaviors: while they seek understanding and support from others, they also fear negative evaluations or social stigmatization. This internal psychological conflict may further influence how adolescents cope with NSSI.

In the dynamic adaptation theoretical model of adolescent NSSI disclosure, the core feature of the second stage is the individual’s psychological conflict between disclosure and non-disclosure. The results of this study indicate that the primary motivation for most adolescents to disclose self-injurious behaviors is the desire to receive emotional support and understanding, as well as to obtain practical assistance at the behavioral level. However, research has found that parental responses often fail to meet adolescents’ psychological needs and may even exacerbate their negative emotional experiences (29). Specifically, adolescents expect their parents to respond with empathy, patience, and acceptance upon learning about their self-injurious behavior, rather than with blame or punishment. Adolescents hope that their parents will not only provide emotional comfort but also take constructive actions to help them better cope with self-injurious behaviors (30). The motivation behind adolescents’ decision to disclose NSSI is fundamentally positive, as they seek to obtain help through the act of disclosure. However, in the actual interaction process, the feedback adolescents receive may significantly deviate from their expectations. This discrepancy between expectation and reality can lead to greater psychological turmoil after disclosure, and may even result in deeper emotional struggles. Some adolescents experience negative emotional feelings after disclosing NSSI. These negative emotional experiences may further diminish their willingness to disclose NSSI in the future, and may cause individuals to repeatedly struggle and vacillate between high expectations and disappointment regarding disclosure.

The results of this study show that in the third stage of disclosure, adolescents choose the specific manner in which to disclose their NSSI behaviors. This manner is generally categorized into active disclosure, indirect expression, or refusal to disclose. In the disclosure action stage, adolescents receive different types of feedback after disclosing their NSSI behaviors to others. In this study, whether through active or passive disclosure, adolescents primarily disclose their self-harming behaviors to parents, friends, peers, and teachers. The research results show that when adolescents’ parents are aware of their NSSI behaviors, fathers’ feedback is generally more lenient than that of mothers. Fathers often show a certain degree of support and understanding, reducing the pressure on adolescents. However, only three data points in this study support this finding, indicating that positive feedback from fathers is relatively rare and not universal. In contrast, mothers’ feedback tends to be more negative, with mothers showing clear signs of anxiety and exhibiting greater misunderstanding of adolescents’ self-harming behaviors. This may be because mothers are more likely to emotionally perceive their children’s self-harming behaviors as “catastrophic events,” which often leads to greater emotional sensitivity and vulnerability when confronting the issue, resulting in criticism and corrective education for the child (31). At the same time, both fathers and mothers exhibit avoidant behaviors in their feedback and reactions, but their ways of expressing this differ. Mothers’ avoidance primarily manifests at the psychological level, where they are unwilling to cognitively accept the reality of their child’s self-harming behavior. In contrast, fathers’ avoidance is more behavioral, such as avoiding the issue by smoking. This difference reflects the varying coping strategies employed by parents when dealing with their child’s NSSI behaviors (32).

The results of this study indicate that adolescents typically receive more positive feedback after disclosing their NSSI behaviors to classmates, friends, and teachers. After becoming aware of adolescents’ self-harming behaviors, classmates, friends, and teachers provide more care and support, while also taking practical steps to discourage adolescents from self-harming again. Compared to parents, classmates, friends, and teachers provide more emotional support and understanding to adolescents with NSSI. Logically, as the closest family members to adolescents, parents should provide more emotional support and understanding. However, the results of this study are exactly the opposite. This may be because parents, when faced with adolescents’ self-harming behaviors, tend to exhibit higher levels of misunderstanding and resistance, failing to consider the issue from the child’s perspective (33). In contrast, classmates, friends, and teachers, as bystanders or peers, are able to understand adolescents’ psychological distress with a more objective and empathetic attitude, thereby providing more targeted support.

The results of this study indicate that in the adjustment phase, adolescents adjust their coping strategies based on others’ feedback and decide whether to continue self-harming behaviors in the future. The nature of the feedback largely influences adolescents’ subsequent attitudes and behavioral patterns toward NSSI. If adolescents primarily receive negative feedback at this stage, they may feel shame, rejection, or helplessness, leading them to hide their self-harming behaviors and reduce their willingness to disclose in the future. However, because their inherent emotional regulation needs have not been met, NSSI behaviors may persist or even worsen (34, 35). Prolonged lack of effective social support may also lead adolescents to develop negative self-perceptions, intensifying feelings of isolation and emotional distress, thereby further reinforcing the occurrence of self-harming behaviors (36). In contrast, if adolescents receive positive feedback in the adjustment phase, they are more likely to reduce the frequency of NSSI and gradually shift towards healthier alternative strategies for emotional regulation. Research suggests that during NSSI interventions, the quality of social support is more critical than the mere level of attention, and only when the responses from supporters meet the psychological needs of adolescents can they truly serve a protective role (37). Therefore, this study further emphasizes the role of others’ feedback in adolescent NSSI behaviors. Although adolescents typically expect to receive help when disclosing NSSI, negative social feedback can not only sustain self-injurious behavior but also affect their future willingness to disclose, thereby increasing psychological distress and the risk of self-harm.

In previous studies on adolescent NSSI disclosure behaviors, some researchers developed measurement questionnaires specifically for NSSI disclosure or conducted quantitative studies using surveys, merely providing a basic exploration of factors influencing adolescent NSSI disclosure (14–16). Although such studies provide important evidence for identifying the risk and protective factors associated with self-injury disclosure, they pay insufficient attention to the dynamic psychological processes underlying this behavior. Some researchers have conducted qualitative studies on NSSI disclosure behaviors, but they did not generate a theoretical model (6, 38). However, this study, employing a grounded theory approach, comprehensively explored adolescent NSSI disclosure behaviors through in-depth interviews and constructed a dynamic adaptation theoretical model of adolescent NSSI disclosure. From a process-oriented perspective, this model reveals how self-injury disclosure behaviors continuously evolve through the interaction of situational triggers, decision-making, actions, and feedback. The model not only integrates the dual influences of individual intrinsic motivations and social feedback but also elucidates the cyclical regulatory mechanisms through which positive and negative feedback maintain or alleviate self-injurious behaviors. Theoretically, the model proposed in this study overcomes the limitations of previous research focused on static factors and introduces a novel framework capable of explaining the dynamic mechanisms underlying adolescents’ NSSI disclosure. This study contributes to a more comprehensive understanding of adolescents’ NSSI disclosure needs, disclosure expectations, and their dynamic processes, offering a novel theoretical perspective for interpreting NSSI disclosure behaviors. Based on the four-stage dynamic adaptation model developed in this study, targeted interventions can be proposed for each distinct stage. For example, during the disclosure decision-making stage, school-based psychological education and peer support programs can help adolescents enhance their self-expression and emotional communication skills, thereby reducing feelings of shame and social isolation. During the disclosure behavior stage, training programs centered on nonjudgmental listening and supportive communication can be provided to parents and teachers to enhance adolescents’ disclosure experiences and their willingness to seek help. Additionally, clinicians can develop assessment tools for self-injury disclosure based on this model to identify individuals at high risk of self-harm.

This study also has several limitations. First, the study recruited adolescents diagnosed with NSSI from psychiatric outpatient clinics, excluding those from community samples, which limits the representativeness of the sample. In future studies, adolescents who engage in self-injury from community samples should be included. Secondly, the study sample was drawn from psychiatric hospitals in China, and its specific cultural context may limit the generalizability of the findings to other healthcare systems or cultural settings. Moreover, as the data rely on participants’ self-reports, there may be some recall bias. Finally, the four-stage theoretical model of dynamic disclosure of non-suicidal self-injury among adolescents developed in this study lacks empirical support from quantitative research, which should be addressed in future studies through quantitative validation.

## 5 Conclusions

The dynamic adaptation model of adolescent NSSI disclosure constructed in this study provides a new theoretical perspective for understanding the disclosure process of adolescent NSSI. This model emphasizes that adolescent NSSI disclosure is not a static, singular behavior, but a dynamic process that continuously adjusts and evolves in response to external feedback. This study provides practical guidance for subsequent interventions by revealing the psychological struggles adolescents experience during disclosure, the influence of social support, and the potential adaptive adjustments following disclosure. It offers theoretical support for the early identification and intervention of NSSI, helping people to gain a more comprehensive understanding of adolescents’ disclosure needs, expectations, and the disclosure process. Furthermore, it provides an important theoretical foundation for the development of more precise and effective intervention strategies in the future.

## Data Availability

The raw data supporting the conclusions of this article will be made available by the authors, without undue reservation.
